# Context-specific targeting of focal adhesion kinase in brain tumors: lessons from glioblastoma and neurofibromatosis type 2-mutant meningioma

**DOI:** 10.3389/fonc.2025.1724278

**Published:** 2025-12-19

**Authors:** Andre Sagerer, Ilker Y. Eyüpoglu, Tareq A. Juratli, Nils Cordes

**Affiliations:** 1 Department of Neurosurgery, Medical Faculty and University Hospital Carl Gustav Carus, TUD Dresden University of Technology, Dresden, Germany; 2 National Center for Tumor Diseases (NCT), NCT/UCC Dresden, a partnership between DKFZ, Faculty of Medicine and University Hospital Carl Gustav Carus, TUD Dresden University of Technology, and Helmholtz-Zentrum Dresden-Rossendorf (HZDR), Dresden, Germany; 3 OncoRay - National Center for Radiation Research in Oncology, Faculty of Medicine Carl Gustav Carus, Technische Universität Dresden, Dresden, Germany; 4 Helmholtz-Zentrum Dresden-Rossendorf, Institute of Radiooncology - OncoRay, Dresden, Germany; 5 German Cancer Consortium (DKTK), Partner Site Dresden, and German Cancer Research Center (DKFZ), Heidelberg, Germany; 6 Department of Radiotherapy and Radiation Oncology, University Hospital Carl Gustav Carus, Technische Universität Dresden, Dresden, Germany

**Keywords:** FAK inhibitor, focal adhesion kinase, glioblastoma, *NF2*-mutant meningioma, pFAK-Y397, precision oncology, synthetic lethality

## Abstract

Focal adhesion kinase (FAK) has long been explored as a therapeutic target in glioblastoma (GBM) based on its overexpression and involvement in invasive signaling. However, clinical trials have consistently failed to show benefit - highlighting a core principle of translational oncology: target presence alone does not imply therapeutic relevance. In contrast, neurofibromatosis type 2 (*NF2*)-mutant meningiomas present a biologically grounded vulnerability, in which loss of the tumor suppressor moesin-ezrin-radixin-like protein (merlin) creates a synthetic lethal dependency on FAK. This context-specific dependency enables clinically meaningful targeting. Early-phase trials already show promising disease control with favorable safety profiles. This mini review examines the contrasting roles of FAK in GBM and *NF2*-mutant meningiomas to underscore the importance of biological context in therapeutic decisions. We propose that *NF2*-mutant meningiomas represent a model for context-specific, synthetic lethal targeting, exemplifying a functional oncogenomics approach to precision oncology.

## 1 Introduction

Translational oncology often faces a fundamental challenge: therapeutic targets are frequently chosen based on expression, therapeutic actionability, and level of awareness rather than functional dependency (1). FAK is a prime example. It has been extensively studied in GBM due to its overexpression and involvement in invasive and survival signaling pathways (2–10). Despite favorable preclinical results, FAK inhibition has not translated into clinical benefit in GBM (11–13), reflecting a broader challenge in validating targets without context-specific functional dependency.

Beyond GBM, it is crucial to consider how other brain tumor entities differ in their biological drivers and therapeutic vulnerabilities. This broader perspective allows a gradual shift from histologic classification toward the molecular mechanisms that define target dependency and the signaling networks closely linked to FAK activity. Within this framework, one of the most prominent alterations involves the *NF2* pathway. The *NF2* gene on chromosome 22q is frequently inactivated in meningiomas by loss-of-function events, such as intragenic deletions and truncating mutations, often together with loss of the remaining 22q arm. Its prevalence scales with World Health Organization (WHO) grade, occurring in 36.8% of WHO grade 1, 60.1% of WHO grade 2, and 69.3% of WHO grade 3 tumors, with enrichment at convexity, parasagittal, and falx locations (14, 15). *NF2*-mutant meningiomas represent a cancer type where FAK inhibition shows actual therapeutic promise (16). These tumors, defined by loss of merlin, exhibit a biologically driven, synthetic lethal dependency on FAK signaling (17–19). What is striking is that this vulnerability remained unnoticed for years - not because it lacked scientific plausibility, but because *NF2*-mutant meningiomas were not prioritized. Their rediscovery as a FAK dependent entity was catalyzed by functional genomics efforts and disease-relevant models alongside mechanistic studies (19, 20). Moreover, the assumption that meningiomas are uniformly benign has contributed to their neglect. Even low-grade *NF2*-mutant meningiomas can encase large vessels and cranial nerves, causing debilitating neurological symptoms and relentless recurrence (20, 21).

Unlike previous reviews that primarily catalog FAK biology and inhibitor development, this work provides a direct mechanistic comparison between GBM and *NF2*-mutant meningioma to illustrate how genetic context - not histological grade or target abundance - dictates therapeutic relevance. We synthesize recent mechanistic and clinical evidence showing that *NF2* loss establishes a synthetic lethal dependency on FAK signaling, in sharp contrast to the non-essential, compensatory role of FAK in GBM. By highlighting this distinction, the review challenges prevailing assumptions about druggability in neuro-oncology and proposes a functional dependency model for target prioritization, biomarker development, and trial design.

## 2 FAK signaling in GBM: presence versus dependency

### 2.1 Clinical and mechanistic rationale for FAK inhibition

In GBM, FAK, encoded by the protein tyrosine kinase 2 (*PTK2*) gene, and its paralog PYK2, encoded by the *PTK2B* gene, are frequently activated downstream of integrin and receptor tyrosine kinase (RTK) signaling, particularly involving the epidermal growth factor receptor (EGFR) and its mutant form *EGFRvIII* (22). This activation is common in tumors with alterations of the tumor suppressor phosphatase and tensin homolog (*PTEN*) and is enriched in invasive and recurrent disease (8, 22). Unlike meningiomas, where *NF2* mutations are common, such mutations are very rare in GBM (< 2%) (23). In GBM, merlin may instead be functionally suppressed, for example by ezrin-mediated sequestration (24), but this does not constitute a recurrent genomic driver.

Thus, FAK is frequently overexpressed in GBM and linked to invasive behavior, glioma stem cell maintenance, and treatment resistance (25–27). These observations initially motivated clinical trials with FAK inhibitors such as GSK2256098, TAE226, and VS-4718 (6, 28, 29). Preclinical studies showed reduced invasion, increased apoptosis and therapy sensitization *in vitro* and in xenografts through pharmacological FAK inactivation (2, 30, 31).

### 2.2 Why FAK failed as a therapeutic target in GBM

However, these results did not translate into improved clinical outcomes (11). Clinical trials in GBM failed to demonstrate significant efficacy of FAK inhibition, revealing critical barriers: functional redundancy in signaling networks (e.g., Src kinase, extracellular signal-regulated kinase (ERK)), and - most importantly - the lack of a predictive biomarker identifying true target dependency (32–34). Such alternative explanations cannot be fully ruled out, as biological and design-related differences between GBM and *NF2*-mutant meningioma may also have contributed to the divergent clinical outcomes (11, 16, 32). Overexpression of FAK alone was insufficient to confer dependency, as GBM cells exhibited marked heterogeneity in their reliance on FAK signaling (19, 35, 36).

This illustrates a broader translational challenge: the assumption that molecular presence equates to therapeutic relevance, which can severely mislead drug development (37–42). In GBM, the disconnect between target expression and mechanistic target addiction exemplifies how the absence of functional context leads to failure - even when the target is biochemically tractable.

### 2.3 Combination therapy strategies and pathway co-targeting

Given the limited efficacy of FAK inhibition as monotherapy, recent efforts in GBM have shifted toward rational combinations that exploit pathway co-dependencies. FAK blockade with Y15 synergizes with temozolomide, increasing apoptosis and reducing intracranial tumor growth (2). In glioma stem-like cells, FAK inhibition enhances sensitivity to EGFR inhibitors via β1-integrin/FAK/EGFR signaling (27). FAK targeting with TAE226 can also radiosensitize a subset of GBM models, though responses are context-dependent (31). A recent chemogenomic screen identified strong synergy between FAK and mitogen-activated protein kinase 1/2 (MEK1/2) inhibition, particularly trametinib, suppressing invasion and tumor burden across heterogeneous stem cell models (43). These data support combination regimens - FAK plus temozolomide, EGFR- or MEK-inhibitors, or radiotherapy - in biomarker-selected patient subsets, with FAK functioning as a signaling amplifier rather than a primary survival dependency.

## 3 FAK as a therapeutic vulnerability in *NF2*-mutant meningioma

### 3.1 Mechanistic basis of FAK dependency through *NF2* loss

Unlike GBM, *NF2*-mutant meningiomas offer a biologically grounded rationale for FAK inhibition (44). The *NF2* gene encodes merlin, which negatively regulates FAK and its downstream signaling via the phosphoinositide 3-kinase/protein kinase B (PI3K/AKT), mitogen-activated protein kinases (MAPK), and hippo/yes-associated protein (hippo/YAP) pathways (45–47). Loss of *NF2* disrupts this control, resulting in constitutive FAK activation and sustained pro-survival signaling (47, 48). This establishes a synthetic lethal relationship: *NF2*-mutant tumor cells exhibit dependency on FAK signaling for survival (49, 50).

### 3.2 Preclinical validation and biological significance

Preclinical studies - employing CRISPR-based screens, phosphoproteomics, and patient-derived models - have consistently demonstrated that FAK inhibition in this context induces apoptosis and suppresses tumor growth (44, 51, 52). This dependency represents a functional vulnerability rather than a correlative finding and may be pharmacologically exploited (53, 54).

**Figure 1:**
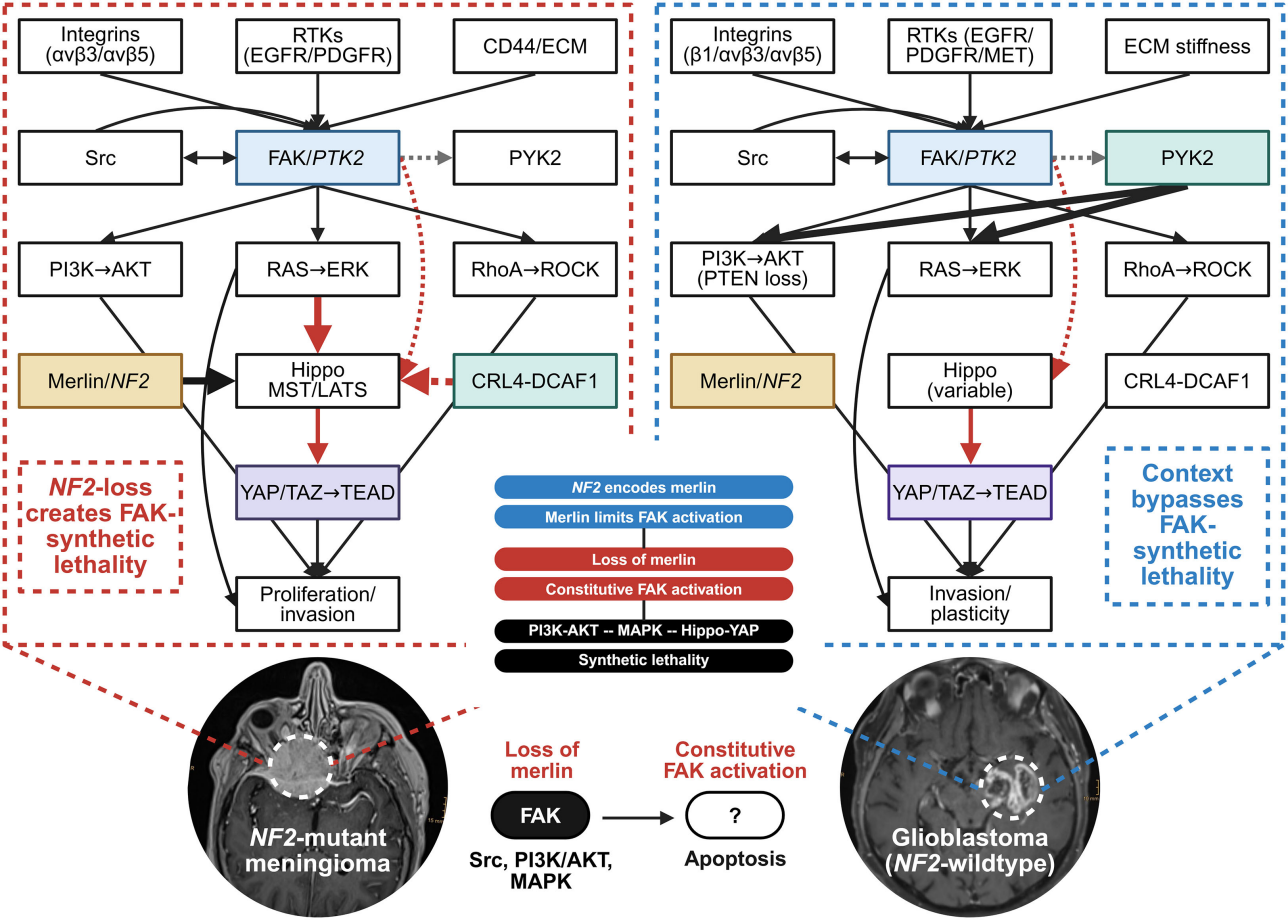
*NF2*-FAK signaling in *NF2*-mutant meningioma and GBM. In *NF2*-mutant meningioma, loss of merlin suppresses hippo kinase activity via CRL4-DCAF1, a cullin-4 RING E3 ubiquitin ligase that uses DCAF1 as its substrate receptor, thereby inhibiting LATS1/2 (large tumor suppressor kinases 1/2) and enabling YAP/TAZ-TEAD (TEA domain transcription factors) dependent transcription, creating FAK dependence. In GBM, *NF2* mutations are rare; instead, integrins, RTKs and ECM stiffness activate FAK and PYK2, with pathway redundancy limiting sensitivity to FAK inhibition. Black solid arrows indicate activation; red solid arrows, inhibition; red dashed arrows, indirect or milieu-mediated suppression; and gray dashed arrows, crosstalk or redundancy. Colors are used solely to improve visual clarity and highlight major signaling components; they do not indicate functional or quantitative relationships. *Created in**BioRender.com* Sagerer, A. (2025) https://BioRender.com/q7fhjqx.

These observations underscore a shift in neuro-oncology: while GBM exemplifies the pitfalls of target selection without functional validation, *NF2*-mutant meningiomas demonstrate how a well-defined molecular context can expose actionable dependencies (47, 55). In this setting, FAK expression indicates a biologically essential role rather than functioning as a passive marker (56). This contrast highlights the importance of functional stratification and serves as a model for precision oncology guided by context (42, 57). The regulatory relationship of FAK dependency in *NF2*-mutant meningioma and GBM is depicted in **Figure 1** (58, 59).

## 4 Clinical evidence: the A071401 trial

### 4.1 The A071401 trial as a context-matched model

The Alliance A071401 trial represents a key inflection point in the translation of FAK inhibition for *NF2*-mutant meningiomas (16). Initially designed as a biomarker-stratified monotherapy study, the trial demonstrated encouraging clinical benefit with the FAK inhibitor GSK2256098. A071401 reported a 6-month progression-free survival (PFS6) of 83% (95% CI 52-98) in WHO grade 1 meningiomas and 33% (95% CI 16-55) in WHO grade 2/3 tumors. Among all patients, 67% experienced disease stabilization, and 3% achieved partial response - indicating meaningful disease control in a historically treatment-resistant setting. The regimen was well tolerated, with most adverse events being grade 1–2 gastrointestinal or dermatologic events, and no treatment-related deaths reported. Moreover, the extracerebral location of meningiomas facilitates better drug exposure, with fewer blood-brain-barrier (BBB) constraints than intra-axial gliomas (60–62). Notably, these results compare favorably to historical controls, where no systemic therapy has consistently improved PFS in recurrent or high-grade meningiomas: recent meta-analytic evidence places median PFS at approximately 3–6 months, with PFS6 rates often below 30% under available salvage treatments (63, 64). These findings must be contextualized within the small, single-arm structure of A071401 and the similarly limited design of prior exploratory meningioma trials (16, 54).

Building on these results, A071401 functions as a genomically guided umbrella platform with parallel targeted arms, including GSK2256098 for *NF2*-mutant and capivasertib for PI3K/AKT-driven tumors. The trial’s adaptive structure not only improves clinical alignment but also provides a framework for stratified intervention in other molecularly defined central nervous system (CNS) tumors (44). *NF2*-mutant meningiomas, once neglected, serve as a prototype for context-informed therapeutic development (65).

### 4.2 Implications for biomarker-driven therapy development

Importantly, genomic co-alterations further refine the therapeutic context. Among these, *CDKN2A/B* (cyclin-dependent kinase inhibitor 2A and 2B) deletions, both heterozygous and homozygous, are strongly associated with shortened time to meningioma progression, with hazard ratios of 5.5 (heterozygous deletion) and 8.4 (homozygous deletion), with prevalence rising by WHO grade and reaching 28.9% homozygous loss in WHO grade 3 tumors (66). In high-grade/progressive meningiomas, the *NF2*-associated genomic subclass frequently harbors *CDKN2A/B* alterations (15). These findings highlight that *NF2* status alone may not be sufficient for patient stratification and provide a rationale to test combination therapies co-targeting FAK in *NF2*-mutant and *CDKN2A/B*-deleted meningiomas. **Table 1** summarizes current and completed key clinical studies conducted within the past fifteen years, exploring FAK targeted therapies with a focus on *NF2*-mutant tumors.

**Table 1:** Prospective clinical studies of FAK targeted therapy in *NF2*-mutant meningiomas and gliomas.

Study / CT identifier / registration region (enrollment years)	Drug	Target	Phase	Randomization	No. of arms	Design	Study centers	Patient population	No. of patients	Results (mOS / mPFS / ORR)	Status	Reference
NCT01138033 / United Kingdom (2010-13)	GSK2256098	FAK	I	No	1	GSK2256098 PK/PD + [11C]−GSK2256098 PET	Multi-center	Recurrent GBM	n=13 (3 dose cohorts; 8 PET substudy)	mOS: NR / mPFS: 5.7 wk (95% CI 3.1-8.3) / ORR: 0% (3/11 SD, 1 patient 11.3 mo)	Completed	PMID: 29788497 (11)
Alliance A071401 / NCT02523014 / United States (2015-17)	GSK2256098	FAK	II	No	1	GSK2256098 mono	Multi-center	Recurrent/progressive *NF2*-mutant meningioma (WHO 1-3)	n=36 (12 WHO 1; 24 WHO 2/3)	mOS: NR (WHO 1); 21.5 mo (WHO 2/3) / mPFS: 12.8 mo (WHO 1); 3.7 mo (WHO 2/3) / ORR: 3% (1 PR)	Completed	PMID: 36288512 (16)
NCI-MATCH Subprotocol U (EAY131-U) / NCT04439331 / United States (2015-17)	Defactinib (VS-6063)	FAK	II	No	1	Defactinib mono	Multi-center	Solid tumors with *NF2* alterations (basket), incl. meningioma	n=33 treated (35 enrolled; 57 *NF2*-altered identified)	mOS: NR / mPFS: 1.9 mo / ORR: 3% (1 PR in choroid meningioma, 40% SD)	Completed	PMID: 39693587 (67)
NCT05798507 / United States (2023-ongoing)	Defactinib or Avutometinib (VS-6766)	FAK, MEK	I	No	2	Single pre-operative dose (PK/PD; tissue concentrations)	Single-center	Newly diagnosed GBM	—	—	Recruiting	ClinicalTrials.gov
5G-RUBY / NCT06630260 / United Kingdom (2024-ongoing)	Avutometinib + Defactinib (± Temozolomide)	MEK + FAK	I/II	Phase 1b non-randomized; phase 2 adaptive	Multiple biomarker arms	Bayesian, open-label platform (e.g., *BRAF*, *NF1*)	Multi-center	Recurrent malignant glioma, incl. GBM	—	—	Recruiting	ClinicalTrials.gov

MEK, Mitogen-activated protein kinase kinase; mOS, Median overall survival; mPFS, Median progression-free survival; NR, Not reached; ORR, Objective response rate; PD, Pharmacodynamics; PK, Pharmacokinetics; PR, Partial response; SD, Stable disease.

### 4.3 Updated clinical landscape and ongoing trials

Beyond A071401, tissue-agnostic trials have exposed the limitations of *NF2* loss as a stand-alone biomarker. In NCI-MATCH Subprotocol U, defactinib achieved a 3% objective response rate (ORR) and median PFS of 1.9 months in *NF2*-altered tumors (67). Similarly, the COMMAND trial showed no benefit from defactinib maintenance in merlin-low mesothelioma (68). In contrast, the FRAME trial demonstrated 42.3% response and 20.1-month median PFS using defactinib plus the RAF-MEK inhibitor avutometinib in MAPK-driven tumors (69), highlighting that pathway-aligned co-targeting may outperform *NF2*-based monotherapy. These findings reinforce that FAK inhibitor success depends on biomarker-guided patient selection and mechanistically grounded combination design.

## 5 Diagnostics and functional tools for therapy selection

### 5.1 Genetic and molecular tools for *NF2* detection

Effective implementation of FAK targeted therapy in *NF2*-mutant meningiomas requires precise diagnostic and stratification strategies (70). The 2021 WHO CNS classification incorporates molecular data into meningioma grading, acknowledging that *NF2* loss and 22q deletion define a distinct biological subgroup, as outlined in cIMPACT-NOW update 8 (71). Routine next-generation sequencing (NGS) or immunohistochemistry (IHC) now provides a practical approach for identifying merlin-deficient tumors in clinical practice, while recognizing that molecular confirmation remains the gold standard (71–74).

### 5.2 Dynamic and surrogate biomarkers for FAK activity

Beyond static genotyping, dynamic functional assessment - particularly phosphorylation of FAK at tyrosine 397 (pFAK-Y397) - has emerged as a key indicator of pathway activation and therapeutic relevance (75, 76). As of 2025, cerebrospinal fluid (CSF)-derived extracellular vesicles (EV) have been explored as an experimental platform (77, 78), for possible future pFAK-Y397 detection. Early studies suggest that serial EV profiling under treatment pressure may correlate with response and progression, but clinical validation is still pending (79–81). This innovation could enhance therapeutic precision by enabling on-treatment decision-making (77).

### 5.3 Digital and AI-based pathology approaches

In parallel, artificial intelligence (AI)-based histopathological tools have matured into clinically applicable decision aids (82, 83). Such deep learning models have shown promising accuracy in predicting *NF2* loss, offering scalable, cost-effective screening in routine pathology or even radiology workflows (84–86). Such tools may be particularly useful in centers without immediate access to molecular diagnostics.

Together, these molecular and digital technologies provide a multilayered framework for stratifying patients based on both genetic status and functional pathway activity - enabling FAK targeted therapy to be deployed where it is most likely to succeed (87–89).

## 6 Combination therapies to enhance FAK inhibition

### 6.1 Mechanistic rationale for combination strategies

Beyond canonical survival signaling, FAK modulates cytoskeletal remodeling, mechanotransduction - the conversion of mechanical cues from the extracellular matrix (ECM) into intracellular signaling - and immune evasion through integrin-mediated adhesion and downstream effectors such as ras homolog family member A (RhoA)/rho-associated coiled-coil-containing protein kinase (ROCK) and YAP and transcriptional coactivator with PDZ-binding motif (YAP/TAZ) (90–93). In *NF2*-mutant settings, FAK inhibition can attenuate YAP/TAZ activity and favor hippo output, leading to downregulation of YAP target genes and induction of apoptosis - especially under high extracellular matrix stiffness or anchorage independent growth, as shown in mesothelioma models (94, 95). While monotherapy with FAK inhibitors has shown promising disease control in *NF2*-mutant meningiomas (16), rational combination strategies are increasingly pursued to enhance efficacy and overcome resistance. Although these concepts derive largely from non-meningioma models (94–100), they represent conserved mechanistic principles that serve here as a hypothesis-generating framework. These combinations aim to intercept parallel survival pathways and exploit synthetic lethal interactions grounded in tumor-specific molecular profiles (57, 101). Furthermore, beyond kinase co-inactivation, recent findings suggest a broader role for FAK in immune modulation. FAK inhibition reduces desmoplasia and reprograms cancer-associated fibroblast (CAF)-rich stroma, increases CD8^+^ cytotoxic T-cell infiltration, and can render tumors responsive to programmed cell death protein 1 (PD-1) blockade (shown in pancreatic ductal adenocarcinoma) (97, 99, 100). It preclinically enables radiotherapy-induced immune priming to unlock checkpoint responses (96, 98).

### 6.2 Opportunities and challenges for clinical translation

However, specific evidence for a combined triple regimen in *NF2*-mutant settings is currently lacking, emphasizing the need for further validation before clinical translation.

The repositioning of FAK inhibitors in *NF2*-mutant meningiomas exemplifies the potential of context-matched therapy; however, key mechanistic questions remain (44). Resistance mechanisms - whether through bypass signaling or stromal compensation - require systematic elucidation through multi-omics, including epigenomics, phosphoproteomics, and single-cell transcriptomics, as well as CRISPR-based screening, whether using drugs, knockdown, or CRISPR technology (52, 53, 102). These technologies can reveal dynamic vulnerabilities and inform next-generation multi-targeting strategies. Integration into multimodal treatment platforms, particularly immunotherapy and radiotherapy, should be prioritized given FAK’s immunomodulatory functions and the immunologically ‘cold’ tumor microenvironment typical of many CNS tumors (98). Furthermore, novel diagnostic tools including emerging molecular positron emission tomography (PET) tracers, CSF-derived EV profiling, and AI-assisted histopathology could, under well supervised conditions, enhance patient selection and treatment monitoring (80, 82, 83, 103). These approaches offer potential for dynamic response assessment and adaptive therapy (57). Ultimately, the insights gained from *NF2*-mutant meningiomas may be generalizable to other tumors (e.g., mesothelioma) with synthetic lethal architectures. Embedding biological context into target selection is essential to achieve therapeutic precision in genetically defined cancer subgroups.

## 7 Discussion

### 7.1 Context-driven target prioritization in neuro-oncology

FAK inhibition in *NF2*-mutant meningiomas represents a rare example of context-defined vulnerability in brain tumors (16, 44). Yet, despite promising early-phase results, key translational barriers remain; *NF2* loss has served as an inclusion criterion and thus an entry point, but remains insufficient to predict response (70). Clinical data (e.g., A071401) reveal variable outcomes, likely influenced by co-alteration (e.g., *CDKN2A/B* or *DMD* (dystrophin) deletions) (66, 102, 104, 105). These may rewire downstream survival pathways and reduce FAK dependence (104). For completeness, FAK has also been implicated preclinically in pediatric brain tumors, including medulloblastoma (106). In GBM, the lack of a consistent FAK dependency likely reflects pronounced cell-state heterogeneity, with transcriptionally distinct subpopulations engaging FAK signaling to different extents (19, 35, 36). As such, *NF2* status should be seen as an entry point - not a definitive biomarker.

From a druggability standpoint, several ATP-binding site FAK inhibitors - including PF-00562271, defactinib, VS-4718 and GSK2256098 - have advanced into early-phase clinical testing with on-target pathway suppression and acceptable tolerability during continuous oral dosing (12, 13). PET-based pharmacokinetic analyses further show that GSK2256098 achieves measurable intratumoral exposure in recurrent GBM despite heterogeneous BBB permeability (11). The A071401 trial additionally supports long-term twice-daily dosing and a favorable therapeutic index in *NF2*-mutant meningioma (16). Together, these data indicate that FAK inhibitors are pharmacologically tractable in brain tumors, with remaining challenges focused mainly on sustaining CNS exposure in intra-axial disease and matching treatment to true FAK dependency.

### 7.2 Outstanding barriers to clinical implementation

pFAK-Y397 is a functional surrogate of FAK activation but lacks validated thresholds or standardized clinical assays (53, 75). Detection via CSF-derived EVs is promising but technically challenging: EV heterogeneity, low abundance, and normalization issues hamper reproducibility (80, 81, 107). To date, no validated, widely adopted platform enables scalable pharmacodynamic monitoring. Exploratory biomarkers include hippo/YAP activity signatures, which are enriched in *NF2*- and YAP1-driven meningiomas (108). Biomechanical stiffness measurements using magnetic resonance elastography correlate with meningioma consistency and may serve as non-invasive functional markers (109, 110). Imaging-based biomarkers such as perfusion magnetic resonance imaging, already used to assess treatment response in gliomas (111), could be integrated with molecular readouts (e.g., *NF2*/hippo status) for multimodal profiling. Resistance mechanisms are poorly understood. Preclinical studies suggest bypass mechanisms involving Src kinase, integrin-linked kinase (ILK)-AKT, and cyclin-dependent kinase activation, including CDK2 and CDK4/6 (112–114). Additionally, YAP/TAZ reactivation via ECM stiffness or cytoskeletal stress may restore downstream signaling despite FAK inhibition (115–117). Without phospho-profiling or single-cell analytics, these adaptations remain undetected in clinical trials.

Mechanistically, FAK integrates adhesion, mechanotransduction, and growth signals - but this centrality invites redundancy (53, 118). In the context of ECM stiffening or hypoxia, FAK independent pathways may be preferentially engaged, limiting therapeutic efficacy (90, 119). Such factors likely vary by tumor grade, location and tumor subtype, meaning FAK dependency is not uniform across *NF2*-mutant meningiomas (47). Operationally, diagnostic implementation is lagging. *NF2* sequencing is inconsistently available outside tertiary centers; pFAK quantification (IHC or EV) is still unstandardized; and AI-based histological tools - though high accuracy in predicting *NF2* loss - lack clinical integration (81, 84). Furthermore, no US Food and Drug Administration-listed companion diagnostic exists to guide FAK targeted therapy.

The extent to which the *NF2*-FAK model can be generalized beyond meningiomas remains uncertain. While mechanistically plausible in other merlin-deficient tumors (e.g., mesothelioma, schwannoma), synthetic lethality has not been robustly demonstrated outside meningiomas (68, 120).

In sum, FAK inhibition in *NF2*-mutant tumors is a model for context-driven therapy. But its broader clinical utility depends on three advances: (a) robust, quantitative biomarkers beyond *NF2* mutation; (aa) resistance profiling under therapeutic pressure; and (aaa) integration of functional and digital diagnostics into real-world care (121).

### 7.3 Strategies to overcome translational barriers

Priorities include standardized pFAK-Y397 assessment or EV-based detection once validated, and adaptive enrichment with on-treatment sampling, circulating tumor DNA (ctDNA) monitoring, and rapid cohort expansion (122). Resistance via Src, CDK, or YAP/TAZ reactivation may necessitate combinations such as FAK plus CDK4/6 or YAP/TAZ inhibition. Companion diagnostics - including AI-based *NF2* prediction or molecular PET tracers - should be co-developed with phase I/II trials to enable regulatory alignment. Without such elements, precision medicine will remain confined to niche settings rather than delivering widely translatable clinical benefits.
